# Cytogenetics and stripe rust resistance of wheat–*Thinopyrum elongatum* hybrid derivatives

**DOI:** 10.1186/s13039-018-0366-4

**Published:** 2018-02-05

**Authors:** Daiyan Li, Dan Long, Tinghui Li, Yanli Wu, Yi Wang, Jian Zeng, Lili Xu, Xing Fan, Lina Sha, Haiqin Zhang, Yonghong Zhou, Houyang Kang

**Affiliations:** 1 Triticeae Research Institute, Sichuan Agricultural University, 211 Huimin Road, Wenjiang, Chengdu, Sichuan 611130 China; 2 College of Resources, Sichuan Agricultural University, 211 Huimin Road, Wenjiang, Chengdu, Sichuan 611130 China

**Keywords:** Genomic in situ hybridization, Stripe rust, *Thinopyrum elongatum*, Hybrid progeny

## Abstract

**Background:**

Amphidiploids generated by distant hybridization are commonly used as genetic bridge to transfer desirable genes from wild wheat species into cultivated wheat. This method is typically used to enhance the resistance of wheat to biotic or abiotic stresses, and to increase crop yield and quality. Tetraploid *Thinopyrum elongatum* exhibits strong adaptability, resistance to stripe rust and *Fusarium* head blight, and tolerance to salt, drought, and cold.

**Results:**

In the present study, we produced hybrid derivatives by crossing and backcrossing the *Triticum durum–Th. elongatum* partial amphidiploid (*Trititrigia* 8801, 2*n* = 6*×* = 42, AABBEE) with wheat cultivars common to the Sichuan Basin. By means of cytogenetic and disease resistance analyses, we identified progeny harboring alien chromosomes and measured their resistance to stripe rust. Hybrid progenies possessed chromosome numbers ranging from 40 to 47 (mean = 42.72), with 40.0% possessing 42 chromosomes. Genomic in situ hybridization revealed that the number of alien chromosomes ranged from 1 to 11. Out of the 50 of analyzed lines, five represented chromosome addition (2*n* = 44 = 42 W + 2E) and other five were chromosome substitution lines (2*n* = 42 = 40 W + 2E). Importantly, a single chromosome derived from wheat–*Th. elongatum* intergenomic Robertsonian translocations chromosome was occurred in 12 lines. Compared with the wheat parental cultivars (‘CN16’ and ‘SM482’), the majority (70%) of the derivative lines were highly resistant to strains of stripe rust pathogen known to be prevalent in China.

**Conclusion:**

The findings suggest that these hybrid-derivative lines with stripe rust resistance could potentially be used as germplasm sources for further wheat improvement.

## Background

Sichuan is the largest wheat-producing region in southwest China, both in cultivatable land area and yield. Although many wheat varieties have been cultivated over the years, Sichuan currently faces a lack of breakthrough varieties that is mirrored in reduced grain quality and resistance to pathogens. Wheat stripe rust is one of the most serious wheat diseases. Therefore, stripe rust resistance gene *Yr26* has been widely used in wheat breeding programs since its discovery in the early 1990s [1]. However, dependence on a single gene can be risky, as the rise of new virulent strains (such as the *Yr26*-virulent race V26/Gui 22) can easily lead to a significant decline in wheat production. *Yr26* virulence may represent a major threat to wheat production in the Sichuan Basin and other regions of China [1]. The use of cultivars harboring novel resistance is an efficient and economical alternative to control wheat stripe rust [2]. The yield and quality of common wheat (*Triticum aestivum* L., 2*n* = 6*×* = 42, AABBDD), which accounts for 95% of domesticated wheat in globally, can be improved by modifications of its genetic background [3]. Wild relatives of common wheat display numerous desirable agronomic traits, including strong adaptability, resistance to biotic and abiotic stress, and high-quality of wheat, and might thus potentially enrich the genetic repertoire of domesticated wheat species [4, 5]. Distant hybridization is a commonly used approach to modify the genetic background of common wheat in order to introduce the beneficial traits of wild species into the genome of domestic wheat [6, 7]. To date, numerous related species from the tribe *Triticeae*, including those from genera *Aegilops*, *Agropyron*, *Dasypyrum*, *Hordeum*, *Leymus*, *Lophopyrum*, *Psathyrostachys*, *Secale*, and *Thinopyrum*, have been successfully crossed with wheat, and the resulting cultivars have contributed significantly to wheat production worldwide [8–13].

*Thinopyrum elongatum* (syn. *Lophopyrum elongatum* or *Agropyron elongatum*) is a perennial herb of the tribe *Triticeae* that plays a prominent role as a gene pool source for genomic improvement of common wheat through hybridization. The taxon contains various cytotypes differing in genomic composition and ploidy level: diploid (2*n* = 2*×* = 14, EE, syn. E^e^E^e^), tetraploid (2*n* = 4*×* = 28, E^e^E^e^E^b^E^b^), hexaploid (2*n* = 6*×* = 42, E^e^E^e^E^b^E^b^E^b^E^b^), and decaploid (2*n* = 10*×* = 70, E^e^E^e^E^b^E^b^E^x^E^x^StStStSt) [14–16]. The E genome of the diploid *Th. elongatum* is the basal genome of the taxon [14, 15]. *Th. elongatum* has many agronomically desirable traits, including high-quality pasture resources, high protein content, and tolerance to salt, drought, and cold [17]. Several significant global advancements in wheat–*Th. elongatum* hybridization have been developed since the beginning of the twentieth century [18]. As example, Li and colleagues synthesized the first common wheat–*Th. elongatum* hybrids in China and developed a series of new wheat cultivars, such as allogeneic octoploid *Trititrigia* Xiaoyan 4, 5 and 6 [19, 20]. Subsequently, the Chinese Spring–*Th. elongatum* addition and substitution lines were successfully bred by Dvorak et al. [21]. Recent studies have uncovered that chromosomes 1E and 7E confer wheat resistance to *Fusarium* head blight (FHB), and the source of resistance was pinpointed to a gene located on chromosome 7ES [22, 23]. Most recently, multiple disease resistance genes were identified in the genus *Thinopyrum* and they were transferred successfully to common wheat, including genes conferring resistance to stem rust (*Sr24*, *Sr25*, *Sr26*, *Sr43,* and *Sr44*), leaf rust (*Lr19*, *Lr24*, *Lr29,* and *Lr38*), stripe rust (*Yr50*) and powdery mildew (*Pm40* and *Pm43*) [24–27].

The hexaploid *Trititrigia* 8801 (2*n* = 6*×* = 42, AABBEE) is a genetically stable partial amphidiploid line induced by hybridization of *Triticum durum* with tetraploid *Th. elongatum* Ae41 (2*n* = 4*×* = 28, E_1_E_1_E_2_E_2_) [28]. It harbors genes of *Th. elongatum* Ae41 that protect against many adverse conditions including stripe rust, powdery mildew, FHB, smut, cold, drought, and salinity [29]. However, owing to its extended growth period and the presence of many alien chromosomes, it is impractical for direct use in the wheat breeding industry. Instead, it is more commonly used as a genetic bridge to transfer alien genes and to create addition, substitution, and translocation lines that can be readily employed in wheat breeding [29]. Moreover, the genetic stability and compensatory effect of translocation lines are superior to those of the addition and substitution lines, thus a translocation line is of potentially greater value in breeding and is more highly regarded by breeders [30].

**Fig. 1:**
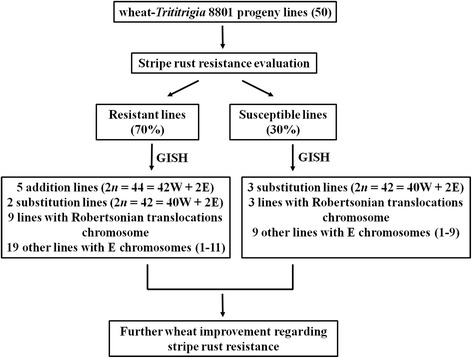
Cytogenetics and stripe rust resistance of wheat–*Thinopyrum elongatum* hybrid derivatives in this study

The objective of the present study was to identify the hybrid progenies derived from crosses between *Trititrigia* 8801 and prevalent *T. aestivum* cultivars grown in the Sichuan region. Using genomic in situ hybridization (GISH) and stripe rust resistance evaluation, we intend to perform a screen to identify genetically stable wheat- *Th. elongatum* progeny lines exhibiting enhanced resistance to stripe rust (Fig. 1). We identified several lines that have the potential to serve as genetic resources for breeding to improve the wheat yield and quality in the Sichuan Basin.

## Methods

### Plant material

The hexaploid *Trititrigia* 8801 (2*n* = 6*×* = 42, AABBEE), which exhibits strong resistance to FHB, stripe rust, and powdery mildew, and wide adaptability including cold, drought, and salt tolerance, was kindly provided by Dr. George Fedak (Eastern Cereal and Oilseed Research Center, Ottawa, Canada). Here we used *T. aestivum* cultivars (2*n* = 6*×* = 42, AABBDD) Shumai482 (SM482) and Chuannong16 (CN16) as the parental genotypes susceptible to stripe rust. Fifty progeny lines were derived by hybridization of SM482 and CN16 with *Trititrigia* 8801 (Table 1). The wheat line SY95–71 was used as a susceptible control in stripe rust resistance tests. For GISH analysis, the Sichuan wheat cultivar J-11 (2*n* = 6*×* = 42, AABBDD) was used to generate blocking DNA, and the total genomic DNA of tetraploid *Th. elongatum* accession PI531750 (2*n* = 4*×* = 28, EEEE) was used to generate probes for labeling.

**Table 1:** Chromosome number and stripe rust response of hybrid progenies

			Chromosome Numbers				Infection type	Resistance/susceptibility
Lines	Materials	Generation	2n	E	W	W/E		
	SY95–71		42		42		4	S
	SM482		42		42		4	S
	CN16		42		42		4	S
	8801		42	14	28		0	R
K13–415-1	8801 × SM482	F_2_	43	5	37	1	4	S
K13–415-2	8801 × SM482	F_2_	42	6	35	1	4	S
K13–415-3	8801 × SM482	F_2_	42	9	33		4	S
K13–415-4	8801 × SM482	F_2_	42	5	37		4	S
K13–422-1	8801 × CN16	F_2_	43	7	36		0	R
K13–437-3	8801/ SM482// SM482	BC_1_F_1_	40	3	37		3	S
K13–437-4	8801/ SM482// SM482	BC_1_F_1_	41	7	33	1	0	R
K13–438-4	8801/ SM482//11 N21	F_1_	40	3	37		0	R
K13–439-1	8801/ SM482// SM969	F_1_	45	3	42		4	S
K13–439-4	8801/ SM482// SM969	F_1_	42	4	38		4	S
K13–440-2	8801/ SM482//N08–51	F_1_	42	3	39		0	R
K14–478-6	8801 × SM482	F_3_	42	5	36	1	0	R
K14–479-4	8801 × SM482	F_3_	44	1	42	1	4	S
K14–480-2	8801 × SM482	F_3_	42	2	40		4	S
K14–485-1	8801 × CN16	F_2_	43	8	35		0	R
K14–490-4	8801 × CN16	F_2_	42	11	31		0	R
K14–499-3	8801/ SM482// SM482	BC_1_F_2_	44	3	41		3	S
K14–500-4	8801/ SM482// SM482	BC_1_F_2_	42	2	39	1	0	R
K14–501-3	8801 / SM482 // 11 N21	F_2_	44	2	42		2	R
K14–501-4	8801 / SM482 // 11 N21	F_2_	42	2	40		1	R
K14–501-5	8801 / SM482 // 11 N21	F_2_	41	1	40		1	R
K14–502-1	8801 / SM482 // 11 N21	F_2_	43	3	40		0	R
K14–502-3	8801 / SM482 // 11 N21	F_2_	43	1	41	1	0	R
K14–502-5	8801 / SM482 // 11 N21	F_2_	47	5	42		0	R
K14–509-1	8801 / SM482 // SM969	F_2_	42	1	41		3	R
K14–511-9	8801 / SM482 // SM51	F_2_	42	1	41		4	S
K14–516-2	8801 / SM482 // SM51	F_2_	44	5	39		0	R
K14–516-4	8801 / SM482 // SM51	F_2_	45	6	39		0	R
K14–528-1	8801 / SM482 F_2_ //SM482	F_1_	45	4	41		4	S
K14–528-4	8801 / SM482 F_2_ //SM482	F_1_	42	2	40		4	S
K14–562-1	8801 / CN16 // CN16	BC_1_F_1_	42	3	39		2	R
K14–637-2	8801 / SM482 // SM482 /// SM482	BC_2_F_1_	43	1	42		0	R
K15–1007-1	8801 × SM482	F_4_	41	4	36	1	1	R
K15–1007-3	8801 × SM482	F_4_	42	5	37		1	R
K15–1007-5	8801 × SM482	F_4_	42	4	37	1	1	R
K15–1016-12	8801 × SM482	F_4_	42	2	40		3	S
K15–1033-8	8801/ SM482 // SM482	BC_1_F_3_	43	3	39	1	0	R
K15–1035-11	8801/ SM482 // 11 N21	F_3_	44	2	42		2	R
K15–1035-12	8801/ SM482 // 11 N21	F_3_	42	2	40		2	R
K15–1035-13	8801/ SM482 // 11 N21	F_3_	44	2	42		2	R
K15–1036-13	8801/ SM482 // 11 N21	F_3_	45	3	42		1	R
K15–1049-1	8801 / SM482 // SM51	F_3_	45	6	39		1	R
K15–1049-4	8801 / SM482 // SM51	F_3_	45	2	42	1	2	R
K15–1048-10	8801 / SM482 // SM51	F_3_	41	4	37		0	R
K15–1058-5	8801 /CN16 //CM104	F_2_	41	1	40		3	S
K15–1020-4	8801 × CN16	F_3_	42	10	31	1	0	R
K15–1053-3	8801 /CN16 // aobaimai3	F_2_	41	1	40		1	R
K15–1083-1	8801/ SM482 // SM482 F_2_ /// SM482	F_1_	44	2	42		1	R
K15–1035-4	8801/ SM482 // 11 N21	F_3_	44	2	42		2	R
K15–1005-3	8801 × SM482	F_4_	42	6	36		1	R

*Abbreviations*: *E* E-genome chromosomes of *Th. elongatum*, *W* A, B, and D-genome chromosomes of wheat, *W/E* translocation chromosome of wheat–*Th. elongatum*. The wheat line SY95–71 was used as a susceptible control. Infection type was based on a scale of 0–4, where 0 = resistant, and 1–4 indicate increasing sporulation and decreasing necrosis or chlorosis, considered highly resistant, resistant, susceptible, and highly susceptible, respectively

### Genomic in situ hybridization

Total genomic DNA of PI531750 and J-11 was isolated using the cetyltrimethylammonium bromide (CTAB) method [31]. PI531750 DNA was labeled with digoxigenin-11-dUTP (or biotin-11-dUTP) by nick translation (Roche, Mannheim, Germany) and used as a probe. Unlabeled J-11 DNA was fragmented to 300–500 base pairs by autoclaving at 100 kPa, 115 °C for 10 min and subsequently was used for a competitor to block A, B, and D-genome sequences from hybridization. Seedling root tips were collected and treated with nitrous oxide for 4 h, 90% acetic acid for 10 min, and digestion by pectinase and cellulase, using the procedure of Komuro et al. [32]. Slides for GISH were prepared as previously described by Han et al. [33], with slight modifications. The hybridization mixture was composed of 37.5% deionized formamide, 15% dextran sulfate, 7.5% 20 × SSC, and 75 μg salmon sperm DNA (initial concentration 10 μg/μL), as well as 50 ng probe DNA (initial concentration 100 ng/μL) and 7.5 μg blocking agent DNA (initial concentration 1250 ng/μL), and the proportion was 1:150 of probe and competitive DNA. A total volume 20 μL of solution was loaded per slide and were denatured together by heating at 80 °C for 5 min, incubated for 8–12 h at 37 °C. Digoxigenin was detected using anti-digoxigenin-rhodamine Fab fragments (Roche, Mannheim, Germany) and biotin was detected with streptavidin-FITC (Roche). Chromosomes were counterstained with 4–6-diamino-2-phenylindole (DAPI) or propidium iodide (PI) solution (Vector Laboratories, Inc., Burlingame, CA, USA). Chromosome images were taken with an Olympus BX-51 microscope equipped with a DP-70 CCD camera. All images were processed using Adobe Photoshop CS 5.0.

### Stripe rust resistance screening

Parental species 8801, SM482 and CN16, derived offspring lines, and the control line SY95–71 were each evaluated for seedling and adult plant responses to stripe rust. Stripe rust tests were performed at the Wenjiang experimental station of Sichuan Agricultural University, Chengdu, Sichuan, China. For each line, plants were grown annually in 2 m rows, with inter-plant spacing of 10 cm and inter-row spacing of 30 cm. The stripe rust susceptible spreader line SY95–71 was planted on both sides of each experimental row. The SY95–71 rows were inoculated at the two-leaf stage with a mixture of fresh urediniospores and talc (1:20 ratio). The *Pst* races (CYR-32, CYR-33, V26/Gui22–9, V26/Gui22–14, Su4, and Su5) were supplied by the Research Institute of Plant Protection, Gansu Academy of Agricultural Sciences, Gansu, China. Stripe rust infection types (IT) were recorded on a scale of 0–4. The plants scored with IT 2 or lower were considered as resistant, whereas the plants with IT 3 or 4 were considered as susceptible. Infection was scored three times, when uniform rust stripe severity was observed on SY95–71 during the booting, flowering, and milky stages [34].

## Results

### GISH analysis of hybrid progenies

Chromosome numbers for 50 randomly selected progeny are presented in Table 1. The mean chromosome number of all hybrid progenies was 2*n* = 42.72, with a range of 40–47. Most lines (94.0%) were 2*n* = 41–45. Of these, progenies with 42 chromosomes were predominant (40%), whereas the remainder of the progeny displayed various degrees of aneuploidy (2*n* = 44, 43, 45, 41, 40, 47) where the order reflects the decreasing proportion of incidence (16.0%, 14.0%, 12.0%, 12.0%, 4.0% and 2.0%). Among these lines with 42 chromosomes, K13–415-3, K14–480-2 and K15–1016-12 derived from the crosses of *Trititrigia* 8801 with SM482 showed the greatest degree of cytological stability. By contrast, some lines exhibited loss of chromosomes, such as K13–415-1 and K15–1007-5 (from 43 to 42). Collectively, these observations indicated that the chromosome number of 8801-derived hybrid progeny tended to conform with the chromosome number of common wheat (2*n* = 42).

**Fig. 2:**
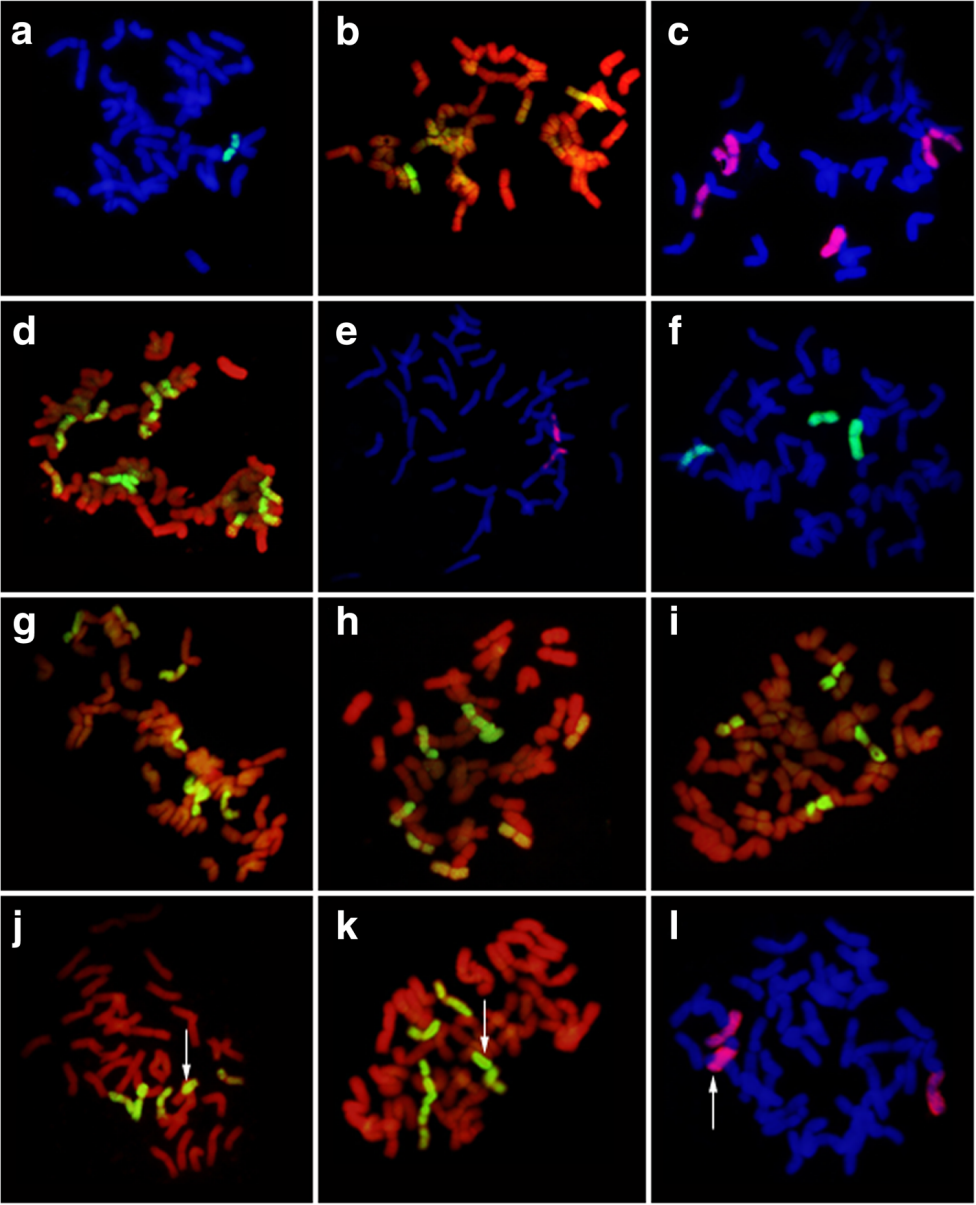
GISH analysis of hybrid progenies at mitotic metaphase. *Thinopyrum elongatum* genomic DNA was used as a probe for in situ hybridization. Chromosomes in red and blue are derived from wheat (W); chromosomes in yellow-green, green, and pink are derived from *Th. elongatum* (E). Arrows indicate Robertsonian translocations. **a** K15–1058-5, 2*n* = 41 = 1E + 40 W; (**b**) K14–480-2, 2*n* = 42 = 2E + 40 W; (**c**) K15–1005-3, 2*n* = 42 = 6E + 36 W; (**d**) K14–490-4, 2*n* = 42 = 11E + 31 W; (**e**) K15–1083-1, 2*n* = 44 = 2E + 42 W; (**f**) K15–1036-13, 2*n* = 45 = 3E + 42 W; (**g**) K13–422-1, 2*n* = 43 = 7E + 36 W; (**h**) K14–485-1, 2*n* = 43 = 8E + 35 W; (**i**) K14–528-1, 2*n* = 45 = 4E + 41 W; (**j**) K15–1007-1, 2*n* = 41 = 4E + 1 W/E + 36 W; (**k**) K14–478-6, 2*n* = 42 = 5E + 1 W/E + 36 W; (**l**) K15–1049-4, 2*n* = 45 = 2E + 1 W/E + 42 W

To identify the alien chromosomes derived from *Trititrigia* 8801 in the 50 progeny lines, we performed GISH using genomic DNA of tetraploid *Th. elongatum* accession PI531750 as a probe. The number of *Th. elongatum* chromosomes in the progeny lines ranged from 1 to 11 (Table 2). Figure 2 provides representative examples of chromosome substitution (Fig. 2a–d), addition (Fig. 2e, f), addition-substitution (Fig. 2g–i), and translocation (Fig. 2j–l) in the progeny. Most of the resulting daughter plants (42%) represent substitution lines, in which from one up to 11 chromosomes of common wheat were replaced by E-specific chromosomes (Fig. 2a–d). Among the lines possessing 42 chromosomes, five exhibited substitution with two E chromosomes (Fig. 2b). Chromosome addition (2*n* > 42) was detected in nine lines, with up to five E chromosomes being added (Fig. 2e, f). Five lines possessed 44 chromosomes, with two additional E chromosomes (Fig. 2e). We also identified eight addition-substitution lines (Fig. 2g–i). In line K13–422-1 (2*n* = 43 = 36 W + 7E), for example, six common wheat chromosomes were replaced and one additional E chromosome was inserted. Most importantly, a single product of intergenomic (wheat–*Th. elongatum*) Robertsonian (Rb) translocation was observed uniformly in 12 progeny lines (Fig. 2j–l). In addition, during the process of selfing (e.g. K13–415-2) or backcrossing (e.g. K13–422-1), majority of the alien chromisomes have been lost and, in some plants, they were completely lacking. The above-mentioned examples indicated that lines with 2*n* = 42 or 2*n* = 44 may be cytologically stable, and those with a Rb translocation may loose the chromosomes in the process of transmission to the next generation, or form a new pair of stable translocation chromosomes by recombination.

**Table 2:** Chromosome constitutions of hybrid progenies

Chromosome number	Number of lines	Number of E chromosomes (number of lines)
40	2	3 (2)
41	6	1 (3), 4 (1), 4.5 (1), 7.5 (1)
42	20	1 (2), 2 (5), 2.5 (1), 3 (2), 4 (1), 4.5 (1), 5 (2), 5.5 (1), 6 (1), 6.5 (1), 9 (1), 10.5 (1), 11 (1)
43	7	1 (1), 1.5 (1), 3 (1), 3.5 (1), 5.5 (1), 7 (1), 8 (1)
44	8	1.5 (1), 2 (5), 3 (1), 5(1)
45	6	2.5 (1), 3 (2), 4 (1), 6 (2)
47	1	5 (1)

### Stripe rust resistance evaluation

**Fig. 3:**
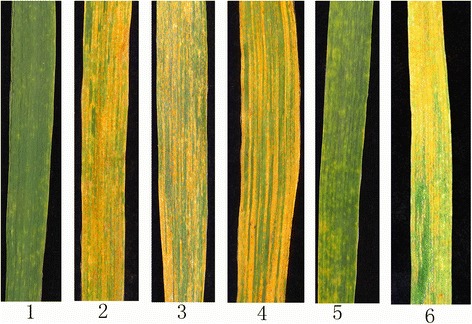
Representative examples of stripe rust resistance in parental lines, hybrid progeny, and controls. (1) *Trititrigia* 8801; (2) *T. aestivum* Shumai482; (3) *T. aestivum* Chuannong16; (4) *T. aestivum* SY95–71; (5) resistant derivative line K15–1007-1; (6) susceptible derivative line K15–1016-12

Each of the 50 derivative lines, the parental lines (8801, SM482 and CN16), and a stripe rust-sensitive control line (SY95–71) was evaluated for stripe rust resistance using a mixture of *Pst* races (see Materials and Methods). Representative examples of stripe rust resistance are provided in Fig. 3. At the seedling and adult plant stages, the 8801 parental line was highly resistant to stripe rust (IT = 0). By contrast, SM482, CN16, and SY95–71 showed IT scores of 4, indicating susceptibility to stripe rust (Table 1). Of the 50 derivative lines, 35 (70.0%) exhibited resistance to stripe rust, whereas the remaining 15 (30%) were susceptible.

## Discussion

Distant hybridization is a promising method to transfer agronomically valuable genes from wild relatives to common wheat. Creating intermediate lines carrying alien chromosomes provides a basis for using germplasm resources of wheat relatives to improve domesticated wheat [35, 36]. *Th. elongatum* harbors many beneficial genes that protect against adverse conditions such as disease, cold, drought, and salinity, and thus represents a promising gene donor to improve tolerance to biotic or abiotic stresses in common wheat [37–39]. In the current study, we produced hybrid progenies by crossing a *Triticum durum–Th. elongatum* partial amphidiploid (*Trititrigia* 8801, 2*n* = 6*×* = 42, AABBEE) with prevalent wheat cultivars grown in the Sichuan area. A previous study demonstrated that the chromosome number of derivative progeny is gradually reaching back the natural chromosome number of *T. aestivum* (2n = 42) with each subsequent generation, and a similar phenomenon has been observed in both inbred and backcross lines [40]. In the present study, we provided another evidence that the number of chromosomes in hybrid progenies approaches 42 (*T. aestivum* L*.*, 2*n* = 42). It is often observed that chromosomes of different parental subgenomes segregate unequally to daughter cells in progeny derived from distant hybridization. Such observation may result from the chromosome elimination during mitosis [41, 42]. Also in our study, we observed unequal chromosome divisions. For example, K13–415-1, K13–415-2, K13–415-3, and K13–415-4 are derived from the same individual parental lines but differed in chromosome number. Likewise, K14–485-1 and K14–490-4 were derived from the same parental lines (*Trititrigia* 8801 and CN16) and also differed in chromosome number.

Identification of alien chromosomes against the wheat chromosome background is an essential step in utilizing alien genetic resources. The Rb translocations typically result from erroneous repair of double strand breaks (by NHEJ pathway), where the p-arms and possibly also one centromere from the original monoarmed chromosome are lost [43]. In the wheat meiosis the process, sister centromeres often lose their coordination in metaphase I and lead to stable bipolar attachment and frequent separation of sister chromatids or to misdivision in the progression of anaphase I, and misdivision may occur across the centromere region or across thepericentric chromatin and subsequent fusion of broken centromeres [44]. In the present study, GISH revealed that all translocated chromosomes had undergone Rb translocation. The line K13–415-1 had a Rb translocation chromosome transmitted to the following generation by single-seed descent, which indicated that this translocation chromosome may remain stable in future generations. Furthermore, some generations derived by single-seed descent from the same parental lines, such as line K13–438-4, showed an elevated number of chromosomes produced by Rb translocations, which indicated that continuous selfing favors the inheritance of alien chromosomes in future generations and contributes to the appearance of new alien types. A similar phenomenon was observed in a previous study by Guan [45]. The formation of a new translocation is probably due to multiple chromosomes breakages in late meiosis at the same time fracture, and chromosomal fragments are misjoined to form aberrant novel chromosome [46]. In addition, the present GISH analysis showed that during the process of selfing or backcrossing, alien chromosomes are predominantly lost or (in some individuals) completely lost. Our findings are in agreement with previous study of Wu et al. [47] in the sense that the plants from the earlier generations tend to sustain/retain more alien chromosomes in comparison to those of later generations. With increase in number of selfing or backcrossing generations, alien chromosome differentiation occurs. In the present study, we identified five relatively stable addition lines (2*n* = 44 = 42 W + 2E) and five substitution lines (2*n* = 42 = 40 W + 2E). Furthermore, a previous study indicated that partial homology exists between wheat and *Th. elongatum* [48], and Cai et al. [49] identified genes that promote partial homologous chromosome pairing in *Th. elongatum*. In addition, in wheat, *Ph1* is a major chromosome pairing locus facilitating correct pairing of homologous, and in wheat hybrids *Ph1* prevents pairing between related chromosomes [35, 50, 51]. This may be another important reason for the chromosome pairing diversity of *Th. elongatum* and different wheat-derived hybrids observed in the present study.

The Sichuan Basin is one of the most important regions for wheat production in China, but it has suffered a serious stripe rust epidemy in recent years. In this study, a series of stripe rust-resistant strains was obtained through distant hybridization with strains carrying E-genome chromosomes. Further work is needed to identify the genomic composition of these lines by fluorescence in situ hybridization (FISH) and to evaluate their agronomic performance. Nevertheless, these lines provide novel and valuable bridge resources for improvement of stripe rust resistance in wheat.

## Conclusion

The use of amphiploid provides a facile method to transfer alien genes to common wheat compared with the direct use of wild species [52]. In this study, we selected and identified substitution, addition, and translocation lines that exhibited high resistance to stripe rust. Although the experimental lines are still in early generation, these lines have the potential to serve as primary material for wheat genetic improvements. Further work is needed to identify the genomic composition of these lines by FISH and to evaluate their agronomic performance. In conclusion, genes with desirable traits were transferred to wheat, thereby extending the genetic source of wheat breeding, enriching genetic diversity and improving the yield and quality of wheat.
